# Anisotropy of the sea surface height wavenumber spectrum from altimeter observations

**DOI:** 10.1038/s41598-019-52328-w

**Published:** 2019-11-04

**Authors:** Shihong Wang, Fangli Qiao, Dejun Dai, Xiaohui Zhou

**Affiliations:** 1 First Institute of Oceanography, Ministry of Natural Resources, Qingdao, China; 2 Laboratory for Regional Oceanography and Numerical Modeling, Qingdao National Laboratory for Marine Science and Technology, Qingdao, China; 3 Key Laboratory of Marine Sciences and Numerical Modeling, Ministry of Natural Resources, Qingdao, China; 4 Graduate School of Oceanography, University of Rhode Island, Kingston, USA

**Keywords:** Earth and environmental sciences, Ocean sciences, Physical oceanography

## Abstract

In this paper, the zonal and meridional sea surface height (SSH) wavenumber spectra are systematically calculated using along-track and gridded altimeter products, and the slopes of the SSH wavenumber spectra over the mesoscale band, which is defined by the characteristic length scale of mesoscale signals, are estimated. The results show that the homogeneous spectral slopes calculated from the along-track and gridded altimeter datasets have a similar spatial pattern, but the spectral slopes from gridded altimeter data are generally steeper than that from the along-track data with an averaged difference of 1.5. Significant differences are found between the zonal and meridional spectra, which suggest that SSH wavenumber spectra are indeed anisotropic. Furthermore, the anisotropy exhibits strong regional contrast: in the equatorial region, the zonal spectrum is steeper than its corresponding meridional spectrum, while in the eastward-flowing high EKE regions the meridional spectrum is steeper than its zonal counterpart. The anisotropy of SSH wavenumber spectral slopes implies that EKE distributes anisotropically in different directions, and this distribution is closely associated with the generation and nonlinear evolution of mesoscale movements.

## Introduction

Satellite altimetric observations reveal that the ocean is full of mesoscale signals on scales of 100 to 300 km^1,2^, and these signals contain the largest percentage of ocean kinetic energy (KE)^3^. Most of the mesoscale signals are explained as eddies, which arise primarily from baroclinic instability^4^ and further grow and evolve nonlinearly. Meanwhile, in strong currents, quasistationary meanders and rings stretching from jets are also taken as mesoscale signals by altimeter observations. In the wavenumber spectrum, the spectral slope over the mesoscale band is the steepest and nearly constant. The sea surface height (SSH) wavenumber spectrum describes how the eddy kinetic energy (EKE) is distributed in wavenumber space and sheds light on the underlying processes of oceanic turbulence; therefore, it is used to infer the dynamics of geostrophic oceanic flows.

In the theory of quasigeostrophic (QG) turbulence, which is driven by large-scale interior potential vorticity (PV) contrasts and not influenced by boundary anomalies, the KE in three-dimensional turbulence follows a k^−3^ power law^5^; thus, under geostrophic balance, the slope of SSH wavenumber spectrum should be k^−5^. In contrast, in the theory of surface quasigeostrophic (SQG) turbulence, which is entirely driven by the density (or potential temperature) anomaly evolution at the boundary, the KE follows a k^−5/3^ power law^6,7^, corresponding to a k^−11/3^ slope of the SSH wavenumber spectrum. However, the real ocean turbulence is more complex than a single QG or SQG model could accurately describe.

The accumulation of high-quality SSH measurements from satellite altimeters also provides a unique tool to explore the wavenumber spectrum on a global scale. Using the along-track dataset, an array of studies estimating the SSH wavenumber spectrum have been carried out^8–11^. Using 7-year raw along-track data from the TOPEX/Poseidon mission, Xu and Fu^12^ conducted a global geographic distribution of SSH wavenumber spectral slopes and found that SSH spectral slopes over a fixed mesoscale band of 70–250 km have regional differences: in highly energetic, predominantly eastward-flowing currents, such as the Kuroshio Extension, Gulf Stream and Antarctic Circumpolar Current (ACC), the SSH spectral slopes are close to or steeper than k^−4^, which is consistent with the work of Le Traon *et al*.^9^. However, in low EKE regions, the SSH spectra are shallower, with slopes between k^−2^ and k^−3^. Zhou *et al*.^13^ revisited the global distribution of the SSH spectral slopes by removing the temporally incoherent signals from the along-track altimeter data and found that the spectral slopes in low EKE regions could exceed k^−3^. Nevertheless, the geographic spatial pattern of spectral slopes reported by Xu and Fu^12^ is still maintained. However, the SSH wavenumber spectra calculations from the along-track data are based on the assumption that mesoscale turbulence is isotropic. Therefore, along-track data are not appropriate for diagnosing the anisotropy of the wavenumber spectrum.

Existing evidence based on direct observations and numerical simulations, however, strongly suggests that mesoscale eddies are zonally or meridionally elongated and have an anisotropic spatial structure, especially in regions with strong background currents^14–16^. The anisotropic characteristics of mesoscale eddies are important in understanding how mesoscale eddies interact among themselves and with currents, which is still unclear. Stewart *et al*.^16^ diagnosed the anisotropy of eddy variability in the global ocean by examining the variance ellipses of velocity fluctuations. At each location, the index of anisotropy is calculated with the time series of geostrophic surface velocities at this point from altimeter observations and a 1/12°global ocean model. Their results show that the eddy variability is anisotropic. Comparing the SSH wavenumber spectrum computed within a subspace, the method in Stewart *et al*. can maintain the local characteristics. However, Stewart *et al*.’s method does not well focus on the mesoscale band because it failed to distinguish the mesoscale fluctuations from total velocity fluctuations. In this paper, we take advantage of the along-track and gridded altimeter products to compute the SSH wavenumber spectra and diagnose its anisotropic characteristics on a global scale. In section 2, details of the data and methods used for computing the SSH wavenumber spectrum are presented. In section 3, the results are shown, including comparisons between the along-track and gridded altimeter data, zonal and meridional spectra are shown. Finally, the key results are summarized and the implications for mesoscale turbulence are discussed.

## Data and Methods

To focus on the mesoscale component of ocean turbulence, we employed the delayed gridded sea level anomaly (SLA) data collected over 25 years from 1993 to 2017 for the wavenumber spectra calculation. The gridded SLA data are from the latest multimission altimeter product DT2014^17^ (ftp.sltac.cls.fr), which is operated by the Data Unification and Altimeter Combination System (DUACS) and distributed by the Copernicus Marine Environment Monitoring Service (CMEMS). The datasets are interpolated to a 1/4° × 1/4°Cartesian grid resolution and daily sampling; therefore, the spatial and temporal resolutions of the DUACS gridded products are imposed by the temporal correlation function used in the mapping procedure, and they do not represent the effective resolution of the products.

The effective spatial resolution of the gridded SLA is mainly affected by the altimeter constellation, reference period and mapping algorithms. Compared with the previous version of DT2010^18^, many improvements have been implemented for DT2014 reprocessing. Four points stand out in DT2014 reprocessing: a new 20-year altimeter reference period replaces the 7-year reference of DT2010; the end of the low-pass filter length (i.e., the minimum wavelength associated with dynamical structures that the altimeter would statistically identify with a signal-to-noise ratio greater than 1) is adjusted from 60 km to 65 km; more accurately defined spatial and temporal correlation scales are used for optimal interpolation map processing; and refined measurement errors composed of an uncorrelated component and an along-track long-wavelength correlated component are used^17^. More details of the mapping process can be found in the handbook of DT2014. All these improvements contribute to reconstructing more accurate mesoscale signals, and the variance in the DT2014 SLA is increased by 5.1% for wavelengths shorter than 250 km, which makes the effective resolution of the DT2014 SLA reach 100 km at mid-latitudes^17^.

To quantitatively demonstrate the effect of gridded processes on spectral analysis, the filtered along-track altimeter data from the 10-yr T/P repeat-orbit, which cover the period from 1992 to 2002, are also used to calculate the homogeneous SSH wavenumber spectra. These data are distributed by AVISO (ftp.aviso.altimetry.fr). The SSH spectral computations are identical to that of Zhou *et al*.^13^, with the exception that the rectangle domain is enlarged to 16° × 16° to keep pace with the gridded data.

**Figure 1:**
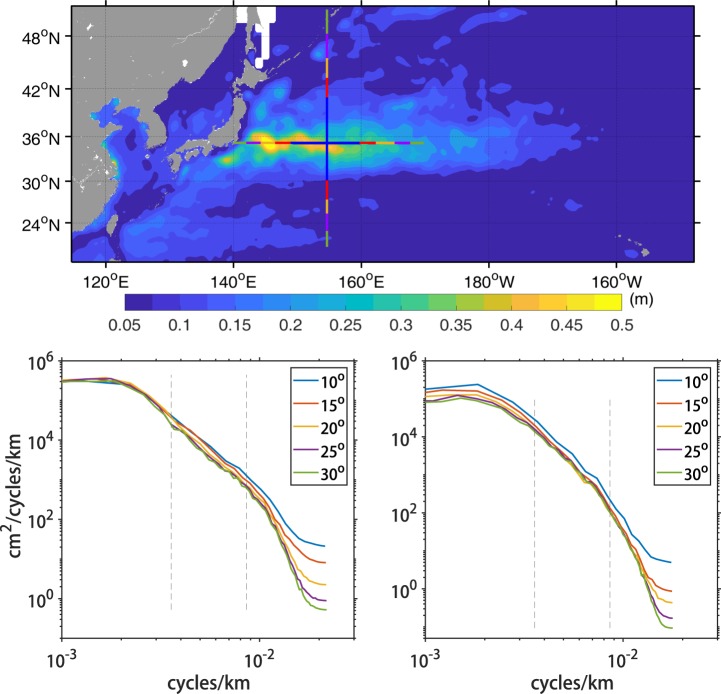
(**a**) Root-mean-square SLA in the Kuroshio Extension based on 25-year gridded SLA. The bold colored lines denote transects used to calculate the wavenumber spectra in (**b**,**c**). (**b**) Time-averaged zonal wavenumber spectra over different zonal transects centered at 35°N, 155°E. (**c**) Time-averaged meridional wavenumber spectra over different meridional transectscenteredat 35°N, 155°E. The vertical dashed lines delineate the mesoscale window of 116–280 km at 35°N.

The fast Fourier transform (FFT)^19^ is used for spectral calculations, which requires that the analyzed data are statistically homogeneous. However, the EKE level in the narrow eastward currents, such as the jet of the Kuroshio Extension and Gulf Stream, is more than ten-fold higher than that in adjacent areas. The spatial series of SSH has a jump at the junction; therefore, the statistical homogeneity is not satisfied. We chose the Kuroshio Extension region (Fig. 1(a)) to evaluate the influence of inhomogeneity on the spectral analysis and to ascertain a suitable transect that both containing the scales of interest and retaining local characteristics well. Note that the wavenumber spectrum computed from the daily SLA is very erratic, especially over small transects (≤10°). The statistically stable wavenumber spectrum can be only obtained through long-term averaging. Figure 1(b) shows the time-averaged zonal and meridional SSH wavenumber spectra centered at 35°N, 155°E over different transects. The results show that while the energy level varies with the choice of transect, the slopes over the mesoscale band are only slightly affected. Given radix 2 FFT, we chose the 16° (containing 64 grids) transect to calculate the SSH wavenumber spectrum in the following analysis. A 10% cosine taper window is applied to the SLA transect to make the boundaries slowly approach zero.

**Figure 2:**
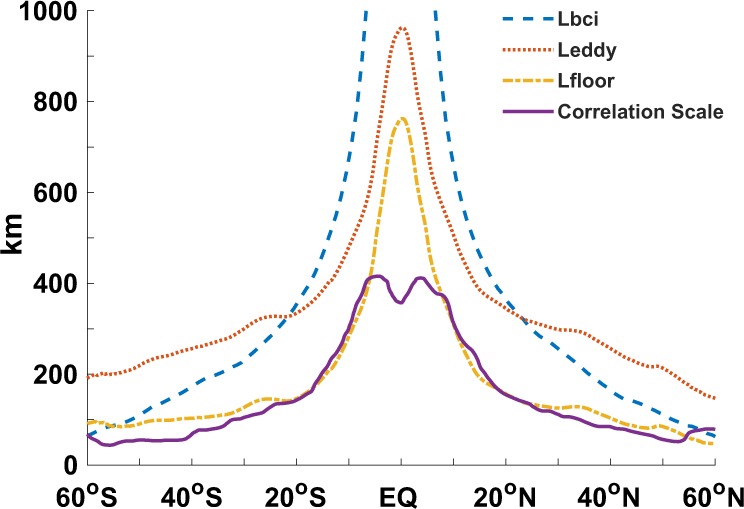
Zonally averaged 2π*L*_*r*_ (dashed curve), where *L*_*r*_ represents the first Rossby deformation radius; the eddy length scale *L*_*eddy*_ (dotted curve) is based on the centroid of KE; the floor of the mesoscale band (dash-dotted curve) and the spatial correlation scale *L*_*correlation_scale*_ (solid curve) used during map processing of DT2014.

Before calculating the SSH wavenumber spectral slopes, the mesoscale band used to estimate the slope must be determined. Xu and Fu^12^ used the fixed mesoscale band of 70–250 km, which was also adopted in subsequent studies^13,20^. However, this band is no longer suitable for gridded data. Since both the Rossby deformation radius^21^ *L*_*r*_ (dashed curve in Fig. 2) and the characteristic length scales of mesoscale eddies^10,11^ *L*_*eddy*_ (dotted curve in Fig. 2) decrease with increasing latitude, the mesoscale band is not dynamically equivalent across all latitudes. Meanwhile, the effective resolution of the gridded data is strongly dependent on latitude, which is closely related to the spatial correlation scale *L*_*correlation_scale*_ (solid curve in Fig. 2) of the altimeter signal^18^ used in the mapping processing. Thus, the mesoscale band used to calculate the spectral slope should vary with changes in latitude. In this paper, we optimized the mesoscale band by defining it with *L*_*eddy*_ as follows:

1 $${L}_{meso\_cap}={L}_{eddy},$$

2 $${L}_{meso\_floor}={L}_{eddy}-200\ast \cos (lat),$$

Various approaches are used to calculate *L*_*eddy*_^22,23^, and Fig. 2 shows the zonal averaged *L*_*eddy*_ given by the centroid method derived from the KE densities in wavenumber space, which can better exclude the influence of the boundary:

3 $${L}_{eddy}=2\pi \frac{\sum KE(K)}{{\sum K\cdot KE(K)}^{}},$$

where KE can be calculated from the SLA under geostrophic balance:

4 $$KE=\frac{{g}^{2}}{2{f}^{2}}[{(\frac{\partial SLA}{\partial x})}^{2}+{(\frac{\partial SLA}{\partial y})}^{2}],$$

To minimize the influence of *L*_*correlation_scale*_, we keep the floor of the mesoscale band larger than the *L*_*correlation_scale*_ at each latitude.

In this study, the homogeneous SSH wavenumber spectra are firstly calculated, and the calculation resolution is 1/4° × 1/4°. For the along-track data, the homogeneous SSH wavenumber spectrum at each grid point was obtained by averaging all the spectra of SSH anomalies along tracks longer than 800 km within a 16° × 16° rectangle box. For the gridded data, the homogeneous SSH wavenumber spectrum is calculated from a rectangle subdomain of SSH through 2-D FFT:

5 $$PP(kx,ky)=fft2(SSH(x,y))$$

And then by summing it up to 1 dimension:

6 $${\rm{P}}({\rm{K}})={\sum }_{K={(k{x}^{2}+k{y}^{2})}^{1/2}}PP(kx,ky)$$

The zonal spectrum for each grid is obtained by averaging all spectra of the zonal SSH anomalies spanning 16° centered at the grid; similarly, the meridional spectrum for each grid is obtained by averaging all the spectra from the meridional SSH anomalies spanning 16° centered at the grid:

7 $${P}_{zonal}(kx)=fft(SSH(x,{y}_{0})),$$

8 $${P}_{meridional}(ky)=fft(SSH({x}_{0},y))$$

The spectral slope is estimated by the least squares of all the wavenumbers falling within the corresponding mesoscale band.

## Results

### Comparison between gridded and along-track data

**Figure 3:**
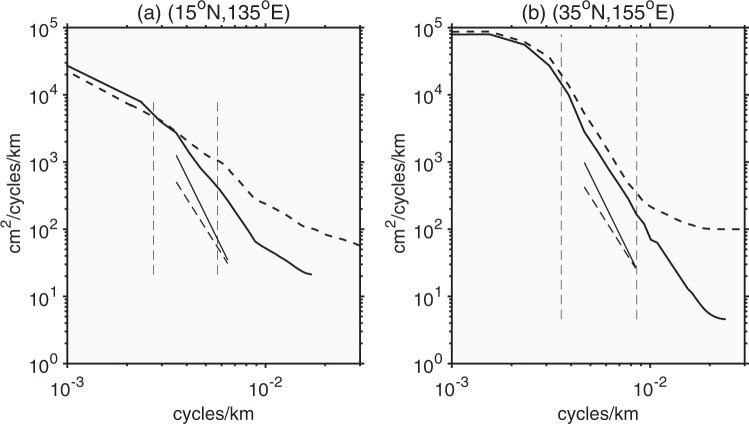
The SSH wavenumber spectra form the along-track (dashed curves) altimeter data and gridded (solid curves) altimeter data centered at 15°N, 135°E and 35°N, 155°E. The solid and dashed oblique lines correspond to slopes of k^−5^ and k^−11/3^, respectively, and the vertical dashed lines delineate the mesoscale bands.

Although many efforts have been made to improve the effective resolution of gridded SLA, the mesoscale signals containing in the along-track data cannot be fully reconstructed. Figure 3 shows the homogeneous spectra computed from the along-track data (hereafter along-track spectra) and gridded data (hereafter gridded spectra) over a 16° × 16° rectangular domain in the North Pacific STCC (15°N, 135°E) and the Kuroshio Extension (35°N, 155°E).The results show that the gridded spectra have remarkably similar shapes at large scales (>300 km) with the along-track spectra. In the STCC, the gridded spectral slope (±standard deviation) over the mesoscale band of 120–320 km is −3.8 ± 0.3, while the along-track spectral slope is −2.7 ± 0.3. In the Kuroshio Extension, the gridded spectral slope over the mesoscale band of 100–223 km is −4.2 ± 0.3 while the along-track spectral slope is −3.9 ± 0.3. Therefore, the spectral slopes of the gridded data are steeper than those of the along-track data.

**Figure 4:**
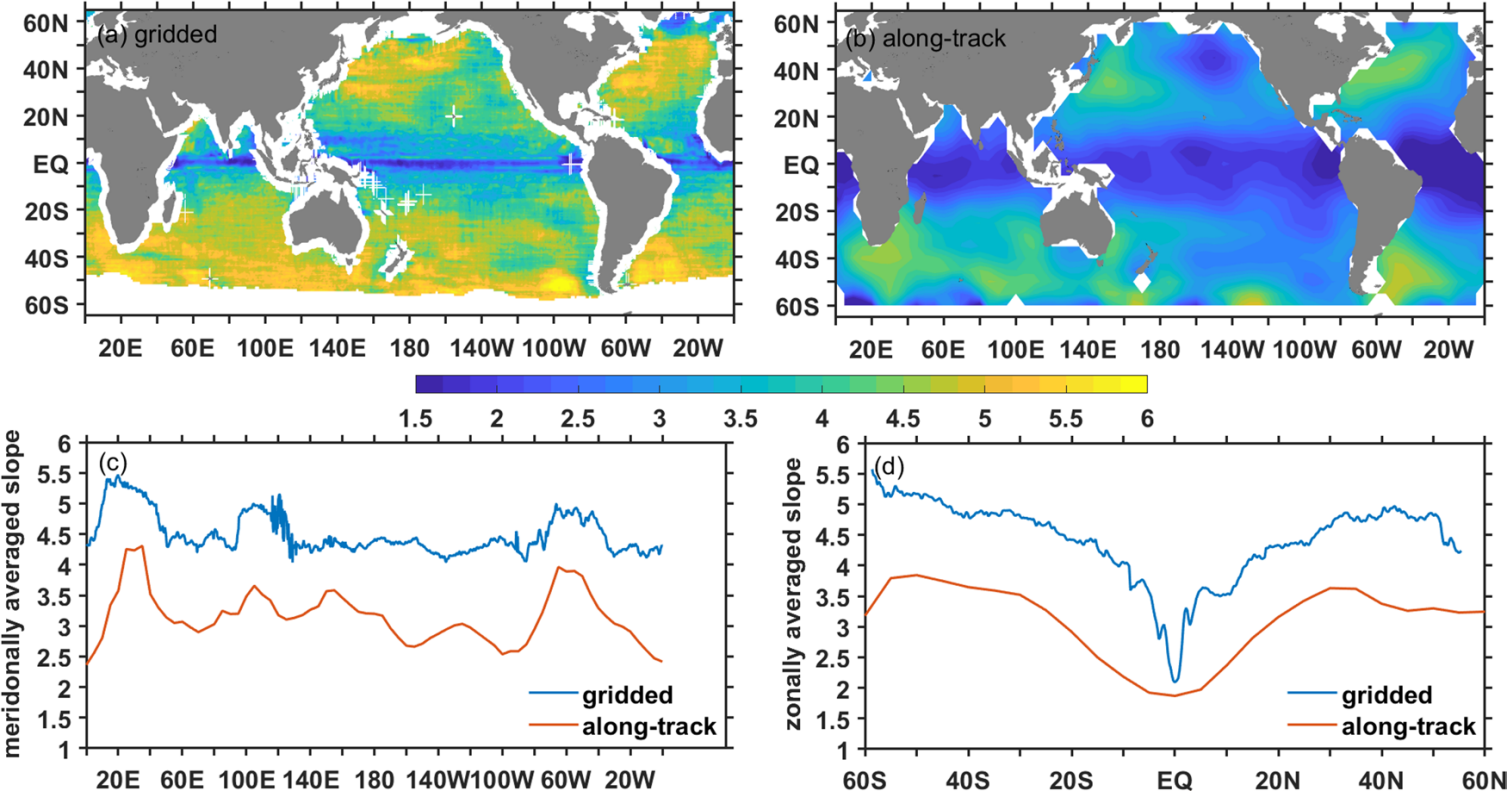
Geographical distributions of homogeneous spectral slopes from gridded altimeter data (**a**) and along-track data (**b**). And the meridionally (**c**) and zonally (**d**) averaged spectral slopes. Note that the sign of the slope is reversed to make the slope values positive.

To investigate whether the difference in steepness before and after the gridding process is general, geographic distributions of the homogeneous spectral slopes from the gridded data and along-track data are displayed in Fig. 4(a,b). The results show that two spatial patterns are very similar with cores of steep slopes in high EKE regions, whereas gridded spectra are generally steeper than the corresponding along-track spectra with a difference about 1.5 in spectral slopes. The zonally and meridionally averaged spectral slopes are also given in Fig. 4(c,d). It shows that while the general patterns of meridionally and zonally averaged along-track and gridded spectral slopes are very consistent, there are some differences in the details, especially in the equatorial and low EKE regions. There are two main factors for the differences: signal loss resulting from the filtering and smoothing in the gridding process and measurement noises in the along-track product. The first steepens the spectrum while the latter shallows the spectrum. However, the values of the gridded spectral slope are well supported by the high-resolution model simulation using realistic atmospheric forcing^20,24^ and *in situ* observations^25^.

### Diagnosis of the anisotropy of SSH wavenumber spectra

**Figure 5:**
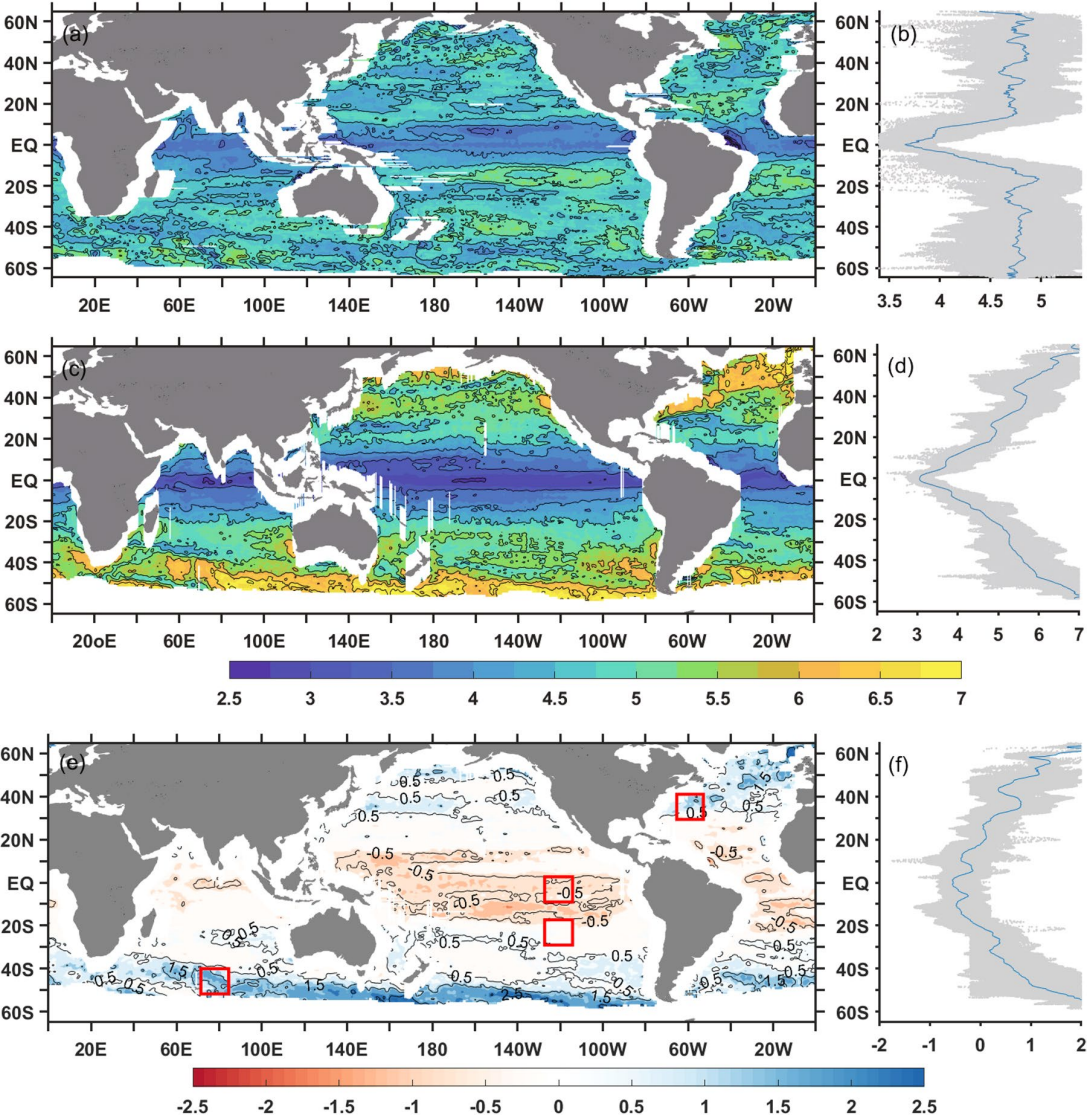
Geographical distributions (left column) and zonal average (right column) of zonal and meridional spectral slopes over the mesoscaleband and their difference. The gray shaded areas in right column indicate the total range. The red boxes denote sub-regions within which zonal and meridional spectral slopes are shown in Fig. 6.

Because the geographical distribution of the gridded spectral slope shares the same spatial pattern with the along-track spectral slope, the zonal and meridional spectra are calculated to diagnose the anisotropic characteristics. Figure 5(a–d) show the global geographic distributions and zonal averages of zonal and meridional spectral slopes. Although both the zonal and meridional spectra slopes are shallower in the equatorial regions than those in any other regions, the pattern of the zonal spectral slope is very different from that of the meridional spectral slope at mid- and high-latitudes, especially in eastward-flowing high EKE currents, such as the Kuroshio Extension, Gulf Stream and ACC. Zonal spectral slopes in these high EKE regions are shallower than those in the surrounding areas, while meridional spectral slopes in these regions are steeper than those in the surrounding areas Furthermore, globally, the meridional spectral slopes are spread over a wider data range (approximately 2.5–7) compared with the zonal spectral slopes (approximately 3.5–5.5). Additionally, the pattern of zonal spectral slopes seems more erratic than that of the meridional slopes and the meridional spectral slopes show better latitudinal dependence and a closer association with the EKE level.

Figure 5(e,f) demonstrate the difference between zonal and meridional spectral slopes specifically. It shows that the global ocean can be categorized into three types of regions. In the equatorial regions (15°S-15°N), denoted by red color, both the zonal and meridional spectral slopes are shallower than those in any other area of the ocean, and the zonal spectra are steeper than the corresponding meridional spectra. The mean values of the zonal and meridional spectral slopes between 15°S and 15°N are 3.9 and 3.1, respectively, and most are even flatter than the predictions of SQG turbulence theories. In the eastward-flowing high EKE currents, including the Kuroshio Extension, Gulf Stream and ACC, which are denoted by blue color, the difference between the zonal and meridional spectral slopes can reach a value of 2, with the meridional spectrum being significantly steeper than its zonal counterpart. The rest of the ocean is quite coincidence with the low EKE regions. In low EKE regions, the differences between the zonal and meridional spectral slopes over the mesoscale band are not significant at less than 0.5.

**Figure 6:**
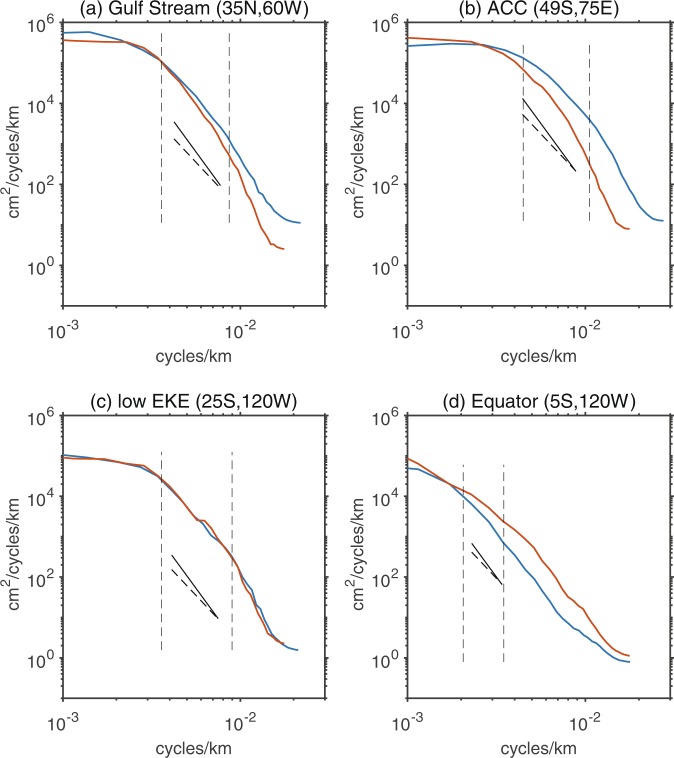
The zonal (blue curves) and meridional (red curves) SSH spectra in four sub-regions denotes. The solid and dashed lines correspond to slopes of k^−5^ and k^−11/3^, respectively, and the vertical dashed lines delineate the mesoscale bands.

To show the difference directly, Fig. 6 shows the zonal and meridional spectra at various latitudes plotted against wavenumber. These spectra were computed over sub-regions denoted by red boxes in Fig. 5(e). Figure 6(a,b) display the estimated zonal and meridional SSH wavenumber spectra in the Gulf Stream (35°N, 60°W) and ACC (49°S, 75°E). The EKE in both eastward-flowing regions is at least one order greater than that in the ocean basin interior^3^. In the Gulf Stream region, the mean zonal SSH spectral slope (±standard deviation) over the mesoscale band of 115–278 km is −4.7 ± 0.3 and the mean meridional slope is −5.4 ± 0.4. In the ACC region, the mean zonal SSH spectral slope over the mesoscale band of 100–223 km is −4.5 ± 0.3 and the mean meridional slope is −5.5 ± 0.4. Although there are differences of exact slope values in different directions and different points, all spectral slopes in high EKE regions are closer to the prediction of QG turbulence theory.

Figure 6(c,d) display the estimated SSH wavenumber spectra in the low EKE region (25°S, 120°W) and tropical ocean interior (5°S, 120°W), respectively. In the low EKE region, the mean zonal spectral slope over the mesoscale band of 124–306 km almost share the same value with the mean meridional spectral slope of −4.3 ± 0.3, which fall within the predicted ranges of SQG and QG turbulence theory. In the tropics (5°S, 120°W), the mean zonal spectral slope over the mesoscale band of 287–486 km is −3.2 ± 0.2 and the mean meridional slope is −3.8 ± 0.2, which is even shallower than the prediction of the SQG turbulence theory. Furthermore, both the zonal and meridional spectra in the tropics (5°S, 120°W) exhibit increasing trends at wavelengths longer than 500 km.

The spectral slope is a manifestation of the generation and evolution of mesoscale signals. Moreover, differences in spectral slopes are directly related with the eddy processes. In the eastward-flowing high EKE regions, isopycnals slope up to the pole, baroclinic instabilities occur in the deep ocean (~1 km) at scales larger than the deformation radius, which can be characterized by an instability in the two-layer model of Phillips^26^ driven by mean quasigeostrophic potential vorticity (QGPV) gradient sign reversal. These mesoscale signals grow rapidly and are long-lasting (e-folding in 1–3 weeks)^23^, during which the strong mean currents can fully stretch and reshape them nonlinearly. Wang *et al*.^27^ further pointed out that the kinetic energy flux over the mesoscale band is anisotropic in these high EKE regions, with more kinetic energy being upscale transferred in the zonal direction. Therefore, more kinetic energy is observed in the zonal direction, which can flatten the zonal spectrum. Additionally, these regions are full of meanders and rings, which are also observed as the mesoscale signals in altimeter observations. These zonally elongated signals also contribute to the flat zonal spectrum.

In tropical regions, however, isopycnals quickly slope up toward the equator and instabilities are dominated by surface intensified modes (~100 to 200 m), which is analogous to the Charney^28,29^ model instability driven by an interaction of the mean surface gradient with a constant interior potential vorticity. This instability makes SQG dynamics more relevant in the equatorial regions; therefore, both zonal and meridional spectra are flatter than that in any other regions. The surface intensified instabilities occur at small scales (about 0.5–1 of the deformation scales) and result in slow growing (e-folding scale of several weeks) mesoscale signals^23^. Furthermore, these mesoscale signals are caught by the Rhines scale^30^ before they are fully developed^31^. Chelton *et al*.^1^ also showed that the number of fully developed mesoscale eddies with characteristic eddy structures is very small throughout the equatorial region. Moreover, laboratory work has demonstrated that young mesoscale signals tend to become meridionally elongated; therefore, more EKE is accumulated in meridional direction and manifests as a flatter meridional spectrum.

In low EKE regions, there is no strong background flow and no surface intensified instabilities, mesoscale signals arise from baroclinic instability then evolve fully. The results of Chelton *et al*.^1^ also show that the majority of mesoscale eddies with mature structures are found in these regions.

With regard to the zonally averaged slopes (Fig. 5(b–d)), the zonal slopes steepen with increasing latitude at low latitudes (15°S-15°N) and keep an almost constant value of approximately 4.7 at mid- and high-latitudes. In contrast, the zonally averaged meridional slopes display a steadily increasing steepness with increasing latitude. In addition to the nonlinear evolution of the mesoscale signals is not anisotropic in zonal and meridional directions, the gridded processes might have an impact on it. Since the satellite orbit has an inclination of about 66°, its ground tracks sample primarily the meridional variability, especially at low latitudes: Therefore, the zonal SSH is more dependent on interpolation.

## Summary and Discussion

In this study, homogeneous SSH wavenumber spectra are calculated over the global ocean from along-track and gridded altimeter data, spectral slopes over the latitude-dependent mesoscale band are estimated. The results show that the slopes of the homogeneous wavenumber spectra are highly correlated with the EKE level. In eastward-flowing high-EKE regions, such as the Kuroshio Extension, Gulf Stream and ACC, the SSH wavenumber spectra slopes are significantly steeper than that in the equatorial regions, which is closely associated with the type of instability. In the tropical regions, Charney instabilities^29^ near the surface are possible, which make the SQG dynamics more relevant. In eastward-flowing high EKE regions, however, Phillips instabilities^26^ are more likely to occur because of the deep zero crossing of the interior QGPV gradient, which make the QG dynamics more relevant. Moreover, the spectral slopes of the gridded altimeter data are generally steeper than those of the along-track altimeter data with a difference of approximately 1.5, especially in the equatorial and low EKE regions, which is mainly because of the loss of mesoscale signals during the gridded processes.

Using the gridded altimeter data, zonal and meridional SSH wavenumber spectra are also systematically calculated on a global scale to diagnose the anisotropic characteristics. The results show that the difference between the zonal and meridional spectral slopes is ubiquitous and has strong regional contrast. In the equatorial region, the zonal spectral slope is generally steeper than the corresponding meridional slope. In contrast, in the eastward-flowing high EKE regions, the zonal spectral slope is significantly shallower than its meridional counterpart. In some areas of the subtropical and subpolar gyre, the difference between the zonal and meridional spectral slopes is not significant and the SSH wavenumber spectrum is almost isotropic. The difference between zonal and meridional spectral slopes is closely related with the nonlinearly evolution of mesoscale signals. In the evolution processes, the meoscale signals interact among themselves and they also interact with the mean currents and the smaller scales nonlinearly and ceaselessly, however, many details of these interactions are still unclear.

The anisotropic SSH wavenumber spectrum strongly suggests that the kinetic energy flux over the mesoscale band is also anisotropic. Using the gridded altimeter data, Scott and Wang^32^ found a universal inverse kinetic energy flux from the deformation scale to larger scales. The inverse kinetic energy flux is considered to accompany the nonlinear interactions among mesoscale signals and between the mesoscale signals and mean currents^23,27^, and the EKE is redistributed in wavenumber space and the spectral slope is changed through these interactions. Wang *et al*.^27^ preliminary stated that in high EKE regions, the altimeter-observed inverse KE cascade is anisotropic with a greater transfer in the zonal direction. Thus, a systematic global survey of the anisotropy of the kinetic energy flux over the mesoscale band should be conducted.

Additionally, certain small-scale or high-frequency ageostrophic motions in the upper layer of the ocean (0–200 m), such as submesoscale processes (O (1–50 km)), waves, and internal tides, can also affect mesoscale turbulence. The submesoscale processes, which are typically characterized by surface frontal structures and the forms of eddies and elongated filaments, can significantly affect the equilibrium state of the mesoscale field^24,33,34^. Qiao *et al*.^35^ also noted that the nonbreaking-wave-induced vertical mixing can generate and enhance turbulence in the upper ocean, with more energy injected in the upper ocean, which could affect the equilibrium state of the ocean. Richman *et al*.^20^ also noted that the presence of strong internal tides could flatten the SSH wavenumber spectrum. Capet *et al*.^36^ noted that in SQG theory, surface KE experiences a clear and significant inverse cascade on a large range of scales (including submesoscale and mesoscale), and the energy source is the ageostrophic component of the flow. This inverse KE cascade can also flatten the KE spectrum. However, energy spectra from *in situ* observations have strong consistency with the predictions of QG theory^25,37–39^. Therefore, the application of SQG to the upper layer of the ocean requires further study, and a full assessment of submesoscale processes, waves, and internal tides in the SSH wavenumber spectrum will require considerable research in the future.

The anisotropy of the SSH wavenumber spectrum also strongly suggests that the characteristic length scale of mesoscale signals is anisotropic. The characteristic length scale of mesoscale signals is often used to determine the magnitude of the lateral turbulence diffusivities^40–42^, such as the isopycnal thickness diffusivity by Gent and McWilliams^43^. Therefore, the specific values of the characteristic eddy length scale affect the simulation of ocean models, especially the coarse resolving ocean model used for climate research. Although considerable work has been done to estimate the characteristic eddy length scale, the anisotropy of the characteristic eddy length scale is still less well known. Eden noted that the characteristic eddy length scale is significantly anisotropic in the North Atlantic Ocean. More accurate characteristic length scales will lead to more accurate values for lateral turbulent diffusivities, which will improve the simulations of ocean models.

As a final note, although evidence of the anisotropy of the SSH wavenumber spectrum is strong, the geographic distribution of spectral slope may be timely. Currently, all altimetric power law results, including those derived from the along-track data, are affected by the compromise in which the measurement noise contributes little to wavelengths longer than 100 km. However, measurement noise is an important factor that influences the difference between the altimeter and *in situ* observation data in spectral analysis. With the Surface Water and Ocean Topography (SWOT) program, the wide-swath satellite will improve the measured SSH resolution potentially down to a spectral wavelength of 15 km^44^, which will enable a more accurate diagnosis of the anisotropy of the SSH wavenumber spectrum. Meanwhile, certain stubborn factors, such as the altimeter constellation and the satellite orbits distribution, may introduce artificial anisotropy to the gridded SLA, which may be embodied in the anisotropy of the wavenumber spectrum.

## References

[1] Chelton DB, Schlax MG, Samelson RM (2011). Global observations of nonlinear mesoscale eddies. Progress in Oceanography.

[2] Chelton DB, Schlax MG, Samelson RM, Szoeke RAD (2007). Global observations of large oceanic eddies. Geophys.res.lett.

[3] Ferrari R, Wunsch C (2008). Ocean Circulation Kinetic Energy: Reservoirs, Sources, and Sinks. Annual Review of Fluid Mechanics.

[4] Smith KS (2007). The geography of linear baroclinic instability in Earth’s oceans. Journal of Marine Research.

[5] Charney, J. G. Geostrophic Turbulence. *J.atmos.sci* **28**, 1/,087-1/,095 (1971).

[6] Blumen W (1978). Uniform potential vorticity flow: Part I. Theory of wave interactions and two-dimensional turbulence. Journal of the Atmospheric Sciences.

[7] Held IM, Garner ST, Swanson KL, Pierrehumbert RT (1995). Surface quasi-geostrophic dynamics. Journal of Fluid Mechanics.

[8] Fu LL (1983). On the wave number spectrum of oceanic mesoscale variability observed by the SEASAT altimeter. Journal of Geophysical Research: Oceans.

[9] Le Traon P-Y, Klein P, Hua BL, Dibarboure G (2008). Do altimeter wavenumber spectra agree with the interior or surface quasigeostrophic theory?. Journal of Physical Oceanography.

[10] Le Traon P-Y, Rouquet MC, Boissier C (1990). Spatial scales of mesoscale variability in the North Atlantic as deduced from Geosat data. Journal of Geophysical Research: Oceans.

[11] Stammer D (1997). Global characteristics of ocean variability estimated from regional TOPEX/POSEIDON altimeter measurements. Journal of Physical Oceanography.

[12] Xu Y, Fu L (2011). Global variability of the wavenumber spectrum of oceanic mesoscale turbulence. Journal of Physical Oceanography.

[13] Zhou XH, Wang DP, Chen D (2015). Global Wavenumber Spectrum with Corrections for Altimeter High-Frequency Noise. Journal of Physical Oceanography.

[14] Huang, H. P., Kaplan, A., Curchitser, E. N. & Maximenko, N. A. The degree of anisotropy for mid-ocean currents from satellite observations and an eddy-permitting model simulation. *Journal of Geophysical Research Oceans* **112** (2007).

[15] Scott RB, Arbic BK, Holland CL, Sen A, Qiu B (2008). Zonal versus meridional velocity variance in satellite observations and realistic and idealized ocean circulation models. Ocean Modelling.

[16] Stewart RH, Shum C, Tapley B, Ji L (1996). Statistics of geostrophic turbulence in the Southern Ocean from satellite altimetry and numerical models. Physica D: Nonlinear Phenomena.

[17] Pujol MI (2016). DUACS DT2014: the new multi-mission altimeter dataset reprocessed over 20 years. Ocean Science Discussions.

[18] Dibarboure G (2011). Jason-2 in DUACS: Updated system description, first tandem results and impact on processing and products. Marine Geodesy.

[19] Cooley JW, Tukey JW (1965). An algorithm for the machine calculation of complex Fourier series. Mathematics of computation.

[20] Richman, J. G., Arbic, B. K., Shriver, J. F., Metzger, E. J. & Wallcraft, A. J. Inferring dynamics from the wavenumber spectra of an eddying global ocean model with embedded tides. *Journal of Geophysical Research Oceans* **117** (2012).

[21] Chelton DB, Deszoeke RA, Schlax MG, El Naggar K, Siwertz N (1998). Geographical variability of the first baroclinic Rossby radius of deformation. Journal of Physical Oceanography.

[22] Eden, C. Eddy length scales in the North Atlantic Ocean. *Journal of Geophysical Research: Oceans* **112** (2007).

[23] Tulloch R, Marshall J, Hill C, Smith KS (2011). Scales, growth rates, and spectral fluxes of baroclinic instability in the ocean. Journal of Physical Oceanography.

[24] Sasaki H, Klein P (2012). SSH Wavenumber Spectra in the North Pacific from a High-Resolution Realistic Simulation. Journal of Physical Oceanography.

[25] Wang D-P, Flagg CN, Donohue K, Rossby HT (2010). Wavenumber spectrum in the Gulf Stream from shipboard ADCP observations and comparison with altimetry measurements. Journal of Physical Oceanography.

[26] Phillips NA (1954). Energy transformations and meridional circulations associated with simple baroclinic waves in a two-level, quasi-geostrophic model. Tellus.

[27] Wang S, Liu Z, Pang C (2015). Geographical distribution and anisotropy of the inverse kinetic energy cascade, and its role in the eddy equilibrium processes. Journal of Geophysical Research: Oceans.

[28] Charney JG (1947). The dynamics of long waves in a baroclinic westerly current. Journal of Meteorology.

[29] Charney JG, Stern M (1962). On the stability of internal baroclinic jets in a rotating atmosphere. Journal of the Atmospheric Sciences.

[30] Rhines PB (1975). Waves and turbulence on a beta-plane. Journal of Fluid Mechanics.

[31] Theiss J (2004). Equatorward energy cascade, critical latitude, and the predominance of cyclonic vortices in geostrophic turbulence. Journal of physical oceanography.

[32] Scott RB, Wang F (2004). Direct Evidence of an Oceanic Inverse Kinetic Energy Cascade from Satellite Altimetry. Journal of Physical Oceanography.

[33] Mcwilliams JC (2016). Submesoscale currents in the ocean. Proceedings. Mathematical, Physical, and Engineering Sciences / The Royal Society.

[34] Sasaki H, Klein P, Sasai Y, Qiu B (2017). Regionality and seasonality of submesoscale and mesoscale turbulence in the North Pacific Ocean. Ocean Dynamics.

[35] Qiao F (2004). Wave-induced mixing in the upper ocean: Distribution and application to a global ocean circulation model. Geophysical Research Letters.

[36] Capet X, Klein P, Hua BL, Lapeyre G, Mcwilliams JC (2008). Surface kinetic energy transfer in surface quasi-geostrophic flows. Journal of Fluid Mechanics.

[37] Bühler O, Callies J, Ferrari R (2014). Wave–vortex decomposition of one-dimensional ship-track data. Journal of Fluid Mechanics.

[38] Callies J, Ferrari R (2013). Interpreting energy and tracer spectra of upper-ocean turbulence in the submesoscale range (1–200 km). Journal of Physical Oceanography.

[39] Rocha CB, Chereskin TK, Gille ST, Menemenlis D (2016). Mesoscale to submesoscale wavenumber spectra in Drake Passage. Journal of Physical Oceanography.

[40] Green J (1970). Transfer properties of the large-scale eddies and the general circulation of the atmosphere. Quarterly Journal of the Royal Meteorological Society.

[41] Stone PH (1972). A simplified radiative-dynamical model for the static stability of rotating atmospheres. Journal of the Atmospheric Sciences.

[42] Thompson AF, Young WR (2006). Scaling baroclinic eddy fluxes: Vortices and energy balance. Journal of physical oceanography.

[43] Gent PR, Mcwilliams JC (1990). Isopycnal mixing in ocean circulation models. Journal of Physical Oceanography.

[44] Qiu Bo, Chen Shuiming, Klein Patrice, Ubelmann Clement, Fu Lee-Lueng, Sasaki Hideharu (2016). Reconstructability of Three-Dimensional Upper-Ocean Circulation from SWOT Sea Surface Height Measurements. Journal of Physical Oceanography.

